# Dilute pilocarpine test for diagnosis of Adie’s tonic pupil

**DOI:** 10.1038/s41598-021-89148-w

**Published:** 2021-05-12

**Authors:** Yung-Ju Yoo, Jeong-Min Hwang, Hee Kyung Yang

**Affiliations:** 1 Department of Ophthalmology, Kangwon National University Graduate School of Medicine, Kangwon National University Hospital, Chuncheon, Korea; 2 Department of Ophthalmology, Seoul National University College of Medicine, Seoul National University Bundang Hospital, 82, Gumi-ro, 173 Beon-gil, Bundang-gu, Seongnam, Gyeonggi-do 13620 Korea

**Keywords:** Diseases, Medical research, Signs and symptoms

## Abstract

We have compared the diagnostic ability of different concentrations of 0.125% and 0.0625% dilute pilocarpine for detecting denervation supersensitivity in unilateral Adie’s tonic pupil. This retrospective, observational, case–control study involved 117 subjects, consisting of 56 patients with unilateral Adie’s tonic pupil and 61 controls with other causes of unilateral dilated pupils. Subjects underwent the dilute pilocarpine test with one of the two concentrations, 0.125% or 0.0625%. Pupillary light reflex was recorded with a dynamic pupillometer at baseline and at 30–40 min after instilling one of the two concentrations of dilute pilocarpine. Diagnostic accuracy of two different concentrations of the dilute pilocarpine test, 0.125% group versus 0.0625% group, were compared by area under the receiver operating characteristic curve (AUC). Diagnostic ability of the dilute pilocarpine test for detecting denervation supersensitivity in unilateral Adie’s tonic pupil was significantly better in the 0.0625% group than in the 0.125% group (AUC = 0.954 vs. 0.840, respectively, *P* = 0.047). In the 0.0625% group, the change in maximal pupil diameter of ≥ 0.5 mm after topical pilocarpine instillation showed 100% sensitivity and 82.8% specificity for detecting Adie’s tonic pupil. This study confirmed that pupillary constriction with 0.0625% pilocarpine is better than 0.125% pilocarpine for detecting denervation supersensitivity in Adie’s tonic pupil. Digital pupillometry is a reliable method for assessing denervation supersensitivity in Adie's tonic pupil.

## Introduction

Dilated pupils result from various factors, such as oculomotor nerve palsy, trauma to the iris sphincter, acute angle closure glaucoma, and pharmacologic inhibition of the parasympathetic pathway^1,2^. Adie’s tonic pupil is caused by damage to the postganglionic parasympathetic nerve of the iris sphincter muscle. It is characterized by loss of direct and indirect pupillary light reflexes, accommodative paresis, segmental palsy of the iris, and denervation supersensitivity^1,3–5^. Denervation supersensitivity is a characteristic sign in Adie’s tonic pupil that is confirmed by pharmacologic testing with a direct-acting weak muscarinic agonist, dilute pilocarpine^1,3,6–8^.

Until now, 0.125% dilute pilocarpine has been generally recommended as the standard pharmacologic agent for demonstrating cholinergic supersensitivity of the iris sphincter^2,4^. However, false positive responses to dilute pilocarpine are not uncommon, where Younge and Buski^9^ found significant constriction in 15% of the normal pupils after instillation of 0.1% pilocarpine. To overcome this limitation, 0.0625% pilocarpine instillation was suggested as an alternative test to detect denervation supersensitivity in Adie’s tonic pupil^10^. However, to the best of our knowledge, no previous study has compared the diagnostic ability of these two concentrations.

A few studies have reported objective quantification of the pupil size in Adie’s tonic pupil^8,11^. Recently, digital pupillometry has been developed, which allows quantification of pupillary light reflex (PLR) parameters in an objective manner^12,13^. Pupillary changes, and in particular, changes in anisocoria, after administration of dilute pilocarpine in unilateral Adie’s tonic pupil can be quantified by digital pupillometry, which can help clinicians distinguish it from other types of physiological and pathological anisocoria.

In the present study, we compared the diagnostic ability of the two concentrations of pilocarpine, 0.125% versus 0.0625%, for detecting denervation supersensitivity in unilateral Adie’s tonic pupil among other causes of unilateral dilated pupils.

## Results

### Baseline characteristics

The demographics and ocular characteristics of patients with unilateral Adie's tonic pupil in the two dosage groups are presented in Table 1. The average age of patients was 44.2 ± 10.9 years in the 0.125% group and 46.6 ± 14.9 years in the 0.0625% group (*P* = 0.489) (Table 1). There were no significant differences in sex, spherical equivalent refractive errors, or best corrected visual acuity between the two groups (Table 1). The frequencies of diabetes mellitus and hypertension were not significantly different between the two groups (all *P* > 0.05).

**Table 1:** Baseline characteristics of patients with unilateral Adie’s tonic pupil in both pilocarpine dosage groups.

Parameters	0.0625% group (n = 22)	0.125% group (n = 34)	*P* value
Age, years	46.6 ± 14.9 (26, 77)	44.2 ± 10.9 (22, 69)	0.489
Sex, male:female	8:14	14:20	0.723
Laterality, right:left	11:11	15:19	0.676
Visual acuity, logMAR (affected eye)	− 0.01 ± 0.14 (− 0.20, 0.30)	− 0.01 ± 0.10 (− 0.20, 0.30)	0.728
Visual acuity, logMAR (unaffected fellow eye)	− 0.04 ± 0.12 (− 0.25, 0.20)	− 0.04 ± 0.11 (− 0.20, 0.30)	0.813
SE refractive errors, D (affected eye)	− 1.28 ± 2.29 (− 7.00, 1.75)	− 1.05 ± 2.90 (− 8.00, 2.88)	0.755
SE refractive errors, D (unaffected fellow eye)	− 1.49 ± 2.03 (− 5.88, 0.25)	− 1.24 ± 2.38 (− 7.13, 1.88)	0.687

D, diopters; logMAR, logarithm of the minimum angle of resolution; SE, spherical equivalent.

Data are mean ± standard deviation (min, max) unless otherwise indicated.

Sixty-one control subjects with other causes of unilateral dilated pupils were included for comparison with a mean age of 49.9 ± 14.4 years; 28 subjects were tested with 0.125% pilocarpine and 33 with 0.0625% pilocarpine. Among control patients, 24 patients (39.3%) were diagnosed with sphincter injury, eighteen (29.5%) with physiologic anisocoria, twelve (19.7%) with oculomotor nerve palsy, four (6.6%) with angle closure glaucoma, two (3.3%) with benign episodic pupillary mydriasis, and one (1.7%) had migraine with episodic unilateral mydriasis. There were no significant differences in age between patients and controls among the different dosagegroups (*P* = 0.052, ANOVA).

### Pupillary light reflex parameters and anisocoria at baseline

The PLR is represented as a response curve of the change in pupil size according to time, and eight parameters are assessed^14^. The pupil diameters are measured at the initial resting state (maximal pupil diameter, mm) and at the peak constricted state after the light stimulus (minimal pupil diameter, mm)^15^. The time interval (in seconds) from the light stimulus to the onset of pupil constriction is recorded as latency. The pupil constriction ratio (%) is the ratio of the reduction in pupil diameter after constriction to the maximal pupil diameter^16^. The rates at which the pupil constricts and dilates are presented as three velocity parameters; average constriction velocity (ACV, mm/s), average dilation velocity (ADV, mm/s), and maximal constriction velocity (MCV)^16^. T75 is the total time for the constricted pupil to recover to 75% of its maximal pupil diameter^16^.

No significant differences were found in baseline PLR parameters between the 0.125% and 0.0625% groups in either the affected or unaffected eye (all *P* > 0.05). The minimal pupil diameter was significantly larger in the affected eye than in the unaffected fellow eye in both groups (both *P* < 0.001). In the constriction phase, the constriction ratio, ACV, and MCV were significantly decreased in the affected eyes of both groups compared to the unaffected fellow eyes (all *P* < 0.001).

**Figure 1:**
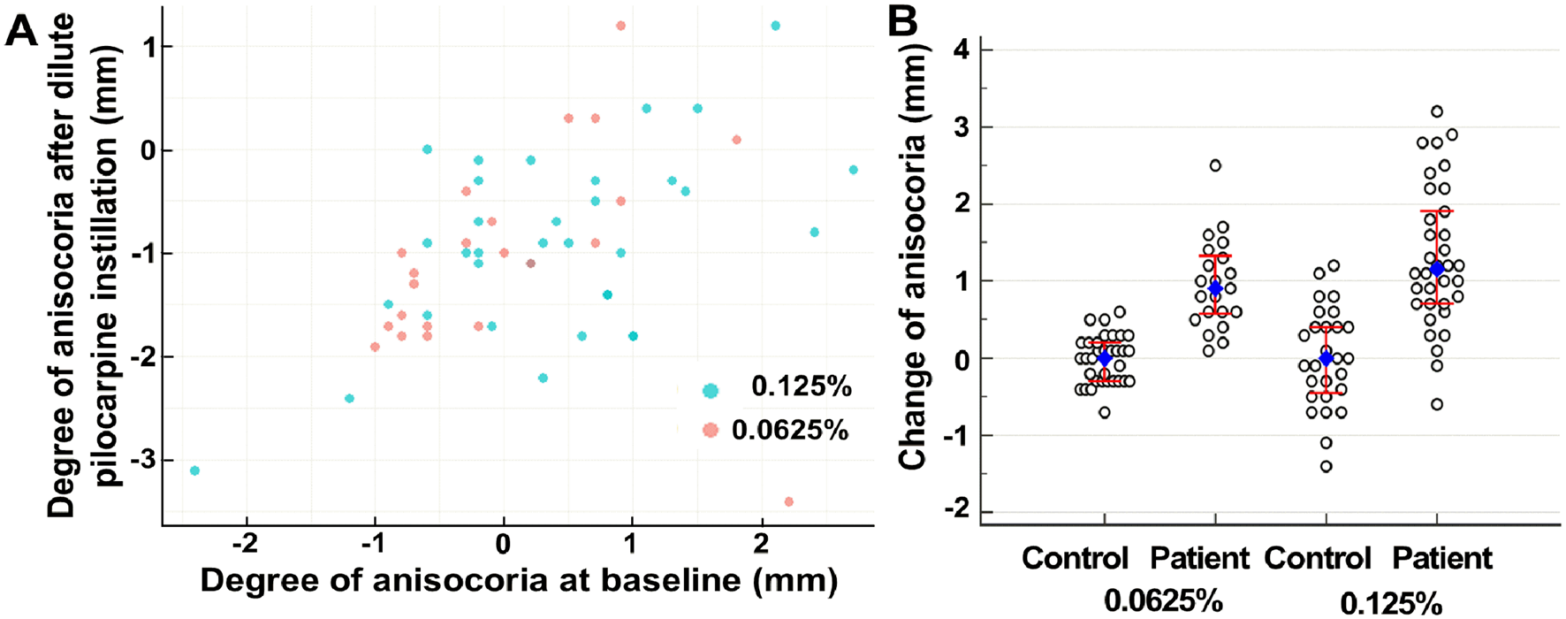
(**A**) Scatter plots of the amount of anisocoria after dilute pilocarpine instillation in patients with unilateral tonic pupil with respect to anisocoria at baseline in both pilocarpine dosage groups. There were no significant differences of ansiocoria in darkness both at baseline (*P* = 0.157) and after pilocarpine instillation (*P *= 0.901) between the two groups. (**B**) Dot plots of the change in anisocoria from baseline to post-pilocarpine test for each patient and control in both pilocarpine dosage groups. The change in anisocoria from baseline to post-pilocarpine test in the patients with unilateral tonic pupil were 1.30 ± 0.92 mm in the 0.125% group and 0.90 ± 0.64 mm in the 0.0625% group showing no significant difference between the two pilocarpine dosage groups (*P* = 0.08). In both pilocarpine dosage groups, comparison of patients with the respective controls revealed significant differences in the amount of change in anisocoria (both *P* < 0.01). The red error bars show 25% and 75% percentiles of the change in anisocoria from baseline to post-pilocarpine test for each patient and control in both pilocarpine dosage groups.

To compare the degree of tonic pupil between the two groups, both the baseline anisocoria at darkness and the change in anisocoria from maximal pupil size to the peak contraction from light stimulus were analyzed. Neither baseline anisocoria at darkness (0.32 ± 0.93 mm in the 0.125% group, and 0.04 ± 0.78 mm in the 0.0625% group, Fig. 1A) nor the change in anisocoria from maximal pupil diameter in darkness to the peak contraction from light stimulus (1.19 ± 0.42 mm in the 0.125% group and 1.26 ± 0.54 mm in the 0.0625% group) showed significant differences between the two pilocarpine dosage groups (*P* = 0.157 and 0.607, respectively).

### Pupillary light reflex parameters after dilute pilocarpine instillation

Table 2 compares the changes from baseline in PLR parameters after dilute pilocarpine instillation measured with digital pupillometry between the 0.125% and 0.0625% groups.

**Table 2:** Comparison of the changes from baseline in pupillary light reflex parameters after dilute pilocarpine instillation measured by digital pupillometry between the two dosage groups.

	0.0625% group (n = 22)	0.125% group (n = 34)	*P* value	Bayes factor
**Affected eye**				
ΔMaximal pupil diameter (mm)	1.33 ± 1.02 (0.5, 5.4)	1.95 ± 0.87 (0.2, 4.2)	**0.018**	3.84
ΔMinimal pupil diameter (mm)	1.22 ± 1.04 (0.4, 5.4)	1.79 ± 0.81 (0.2, 3.9)	**0.031**	3.11
ΔPupil constriction ratio (%)	1.1 ± 2.4 (− 4, 9)	0.6 ± 3.1 (− 13, 5)	0.462	0.28
ΔLatency (s)	− 0.02 ± 0.18 (− 0.4, 0.5)	− 0.04 ± 0.23 (− 0.4, 0.8)	0.658	0.28
ΔACV (mm/s)	0.19 ± 0.34 (− 0.30, 1.50)	0.28 ± 0.38 (− 0.30, 1.20)	0.362	0.41
ΔMCV (mm/s)	0.28 ± 0.46 (− 0.30, 1.80)	0.33 ± 0.47 (− 0.60, 1.50)	0.703	0.29
ΔADV (mm/s)	0.08 ± 0.14 (− 0.10, 0.50)	0.08 ± 0.13 (− 0.20, 0.30)	0.981	0.27
ΔT75 (s)	− 0.02 ± 1.19 (− 2.5, 1.5)	− 0.16 ± 0.98 (− 1.6, 2.9)	0.635	0.31
**Unaffected fellow eye**				
ΔMaximal pupil diameter (mm)	0.27 ± 0.25 (− 0.2, 0.6)	0.68 ± 0.62 (− 0.3, 2.8)	** < 0.01**	10.87
ΔMinimal pupil diameter (mm)	0.13 ± 0.23 (− 0.4, 0.5)	0.39 ± 0.46 (− 0.3, 1.9)	**0.012**	8.04
ΔPupil constriction ratio (%)	1.4 ± 2.8 (− 2, 8)	2.5 ± 4.0 (− 5, 15)	0.233	0.33
ΔLatency (s)	− 0.04 ± 0.02 (− 0.10, 0.0)	− 0.03 ± 0.02 (− 0.10, 0.0)	0.781	0.59
ΔACV (mm/s)	0.23 ± 0.40 (− 0.50, 1.20)	0.36 ± 0.41 (− 0.30, 1.50)	0.249	0.48
ΔMCV (mm/s)	0.42 ± 0.54 (− 0.40, 1.80)	0.50 ± 0.65 (− 0.70, 2.00)	0.625	0.87
ΔADV (mm/s)	0.10 ± 0.13 (− 0.10, 0.30)	0.19 ± 0.23 (− 0.30, 0.60)	0.063	0.31
ΔT75 (s)	0.23 ± 0.94 (− 1.9, 2.4)	0.04 ± 0.82 (− 1.6, 1.7)	0.471	0.29

ACV, Average constriction velocity; ADV, Average dilation velocity; MCV, Maximal constriction velocity; T75, Total time from the peak of constriction to the recovery of the pupil to 75% of maximal pupil diameter; Δ, baseline value minus post-pilocarpine test value.

Data are mean ± standard deviation (min, max) unless otherwise indicated. *P* values in boldface indicate statistical significance.

The change in anisocoria from baseline to post-pilocarpine test was 1.30 ± 0.92 (range, − 0.6–3.2 mm) in the 0.125% group and 0.90 ± 0.64 (range, 0–2.6 mm) in the 0.0625% group (*P* = 0.08 [Bayes factor (BF) = 1.02], Fig. 1B). In both 0.125% and 0.0625% groups, the change in anisocoria from baseline to post-pilocarpine test was significantly larger in the patients than that of controls (both *P* < 0.01 and BF > 300, Fig. 1B).

**Figure 2:**
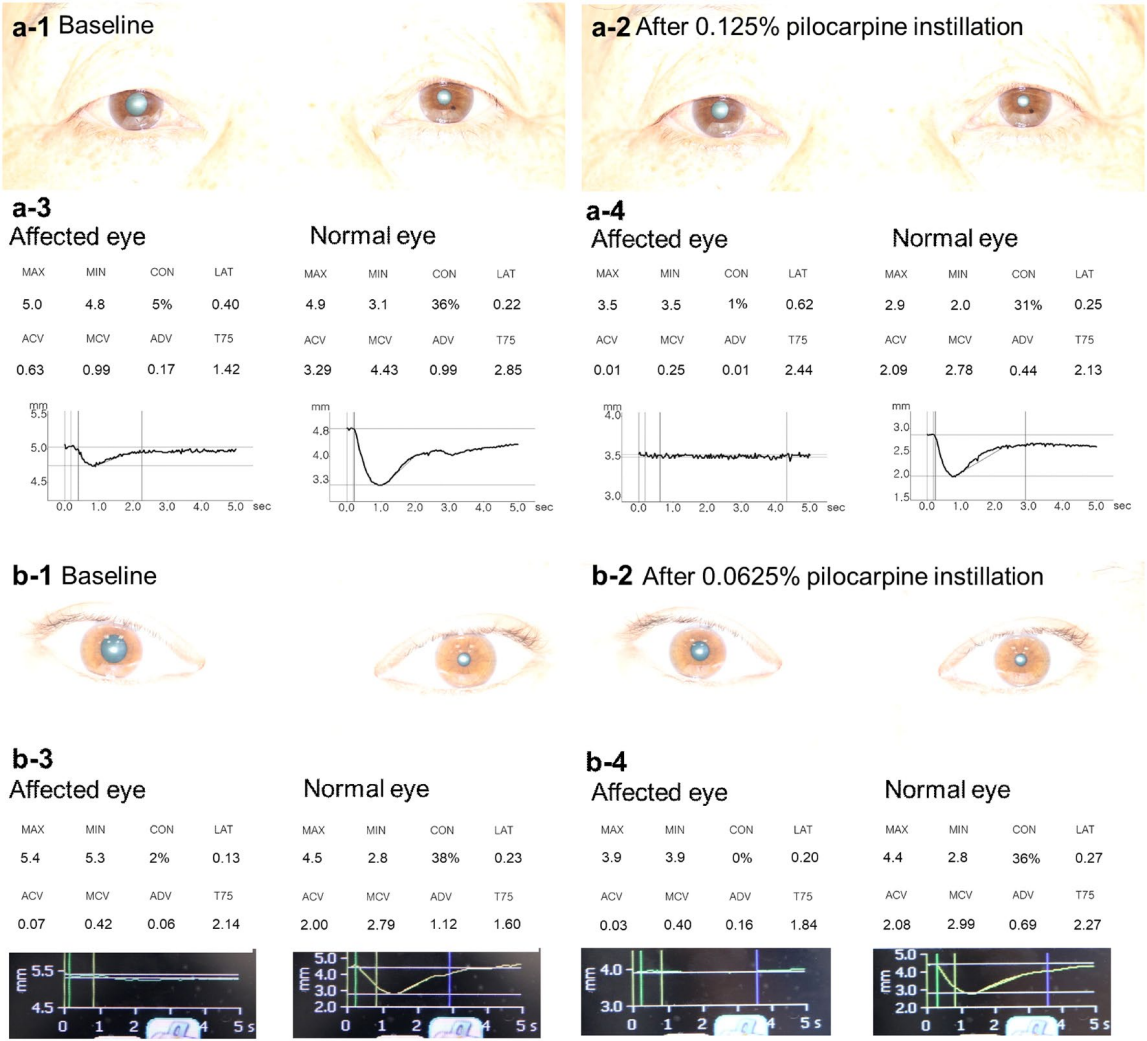
Representative cases of dilute pilocarpine test in unilateral Adie's tonic pupil. The pupillary light reflex (PLR) was recorded with a digital pupillometer. Pupil photographs were taken at baseline (a-1 and b-1) and 30–40 min after pharmacological testing (a-2 and b-2). Both patients at baseline showed mydriasis in the right eye (a-1 and b-1). Eight pupillary light reflex parameters were presented with pupil response curves at baseline (a-3 and b-3) and after 0.125% pilocarpine (a-4) and 0.0625% pilocarpine (b-4) instillation. Thirty minutes after instillation of 0.125% pilocarpine in both eyes, maximal and minimal pupil sizes reduced by 1.5 and 1.3 mm in the affected eye and 2.0 and 1.1 mm in the normal fellow eye (a-3 and a-4). In the patient who underwent 0.0625% pilocarpine testing, the normal fellow eye showed minimal change of maximal and minimal pupil diameters (0.1 mm and no change, respectively) (b-3 and b-4). On the contrary, maximal and minimal pupil diameters of the affected eye reduced by 1.5 and 1.4 mm, respectively. Note that the amount of change in anisocoria from baseline to post-pilocarpine test was 0.2 mm after instillation of 0.125% pilocarpine (**a**) and 1.4 mm after 0.0625% pilocarpine instillation (**b**). ACV = Average constriction velocity; ADV = Average dilation velocity; CON = Pupil constriction ratio; LAT = latency; MAX = maximal pupil diameter; MCV = Maximal constriction velocity; MIN = Minimal pupil diameter; T75 = Total time from the peak of constriction to the recovery of the pupil to 75% of maximal pupil diameter.

Regarding false positive changes in the maximal pupil diameter of the unaffected fellow eye after dilute pilocarpine instillation, 41.2% (14/34) of the 0.125% group showed a decrease of more than 0.6 mm (range, 0–2.8 mm). Conversely, in the 0.0625% group, no subject experienced a decrease or more than 0.6 mm in the maximal pupil diameter of the unaffected fellow eye (range, − 0.2–0.6 mm) (*P* = 0.001). Figure 2 shows representative cases of the dilute pilocarpine test in patients with unilateral Adie's tonic pupil in each dosage group. A considerable amount of pupil constriction in the unaffected fellow eye is shown only after 0.125% pilocarpine instillation, whereas minimal change of pupil diameters are found in the fellow eye after 0.0625% pilocarpine instillation.

### Diagnostic ability of two concentrations of pilocarpine for Adie’s tonic pupil

Comparison of the maximal and minimal pupil diameters of the affected eye at baseline revealed no significant differences between controls and patients with Adie's tonic pupil (*P* = 0.340 [BF = 0.44] and 0.152 [BF = 0.39], respectively, for the 0.125% group, and *P* = 0.503 [BF = 0.23] and 0.058 [BF = 1.47], respectively, for the 0.0625% group).

**Figure 3:**
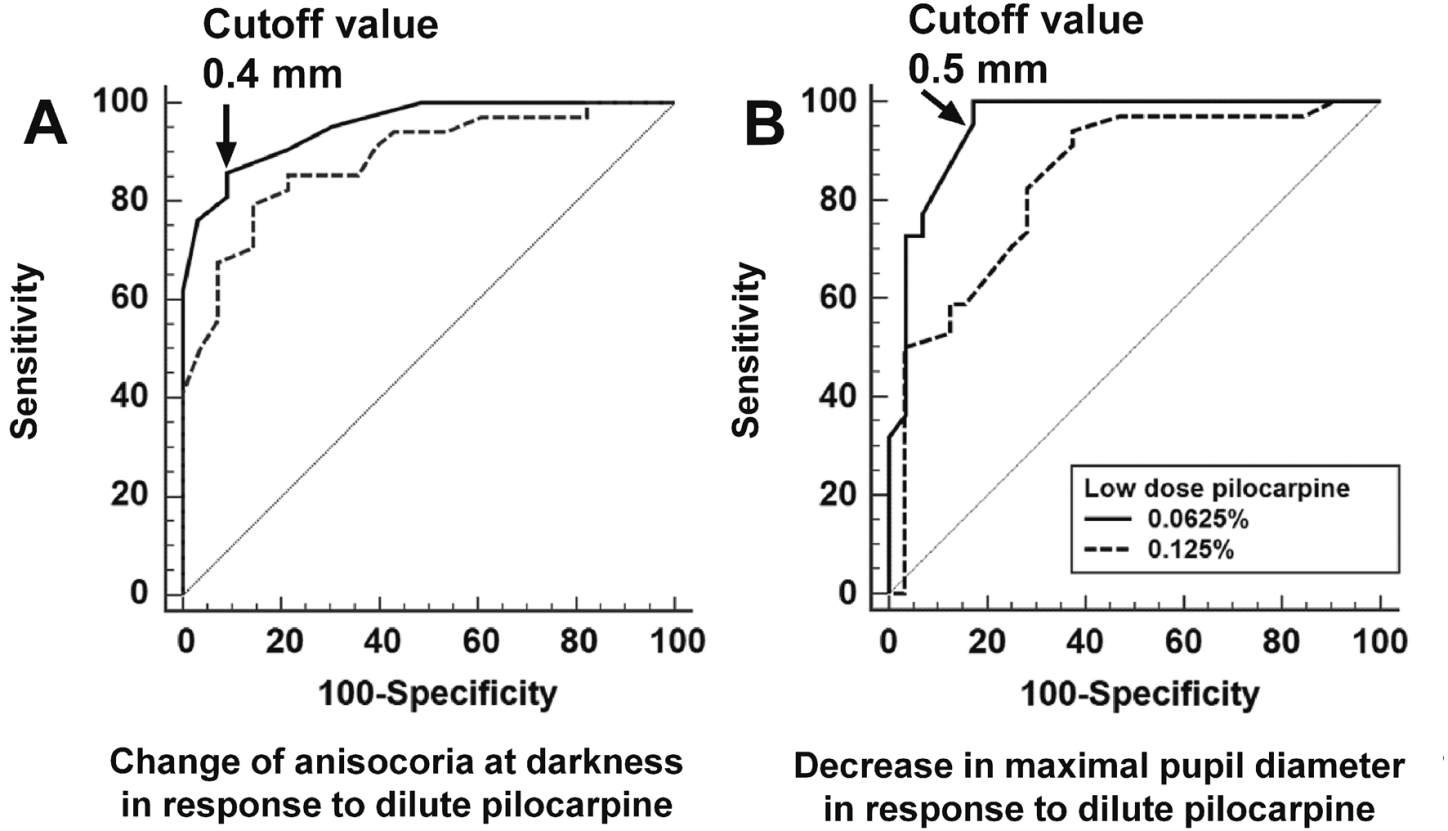
Comparison of the diagnostic ability of two concentrations of dilute pilocarpine for detecting unilateral Adie’s tonic pupil. (**A**) The area under the curve (AUC) of the change in anisocoria at darkness in response to pilocarpine instillation was 0.884 (95% confidence interval [CI], 0.778–0.952) in the 0.125% group and 0.952 (95% CI, 0.857–0.992) in the 0.0625% group (*P* = 0.168). In the 0.0625% group, a change in anisocoria ≧ 0.4 mm after topical pilocarpine instillation showed a sensitivity of 85.7% and a specificity of 90.9% for detecting unilateral Adie’s tonic pupil. (**B**) The AUC of the decrease in maximal pupil diameter in response to pilocarpine instillation was 0.840 (95% CI, 0.729–0.919) in the 0.125% group and 0.954 (95% CI, 0.855–0.993) in the 0.0625% group. The diagnostic efficacy of dilute pilocarpine for detecting unilateral Adie’s tonic pupil using a 0.0625% concentration was better than using a 0.125% concentration (*P* = 0.047), with a cutoff value of ≧ 0.5 mm pupillary constriction after pilocarpine instillation.

The diagnostic performance of the PLR parameters after dilute pilocarpine test was assessed using the area under the curve (AUC). The change in anisocoria at darkness from baseline to post-pilocarpine instillation were compared. The AUC value of the change in anisocoria from baseline to post-pilocarpine instillation was 0.884 (95% confidence interval [CI], 0.778–0.952) in the 0.125% group and 0.952 (95% CI, 0.857–0.992) in the 0.0625% group (Fig. 3A) (*P* = 0.168). In the 0.0625% group, a change in anisocoria ≧ 0.4 mm after topical pilocarpine instillation showed a sensitivity of 85.7% and a specificity of 90.9% for detecting unilateral Adie’s tonic pupil.

The decrease in maximal pupil diameter of the affected eye after dilute pilocarpine administration showed AUC values of 0.840 (95% CI, 0.729–0.919) in the 0.125% group and 0.954 (95% CI, 0.855–0.993) in the 0.0625% group for diagnosing Adie’s tonic pupil, and the difference between the two groups was significant (*P* = 0.047) (Fig. 3B). In the 0.0625% group, a change in maximal pupil diameter ≧ 0.5 mm after topical pilocarpine instillation showed a sensitivity of 100% and a specificity of 82.8% for detecting unilateral Adie’s tonic pupil.

## Discussion

In this study, we compared the diagnostic efficacy of the dilute pilocarpine test performed at two concentrations, 0.125% and 0.0625%, through quantitative analysis of anisocoria and the maximal and minimal pupil diameters. A monocular recording portable digital pupillometer was used to record baseline dark-adapted sizes of the pupil and the dynamics of pupil contraction and dilation in response to a light stimulus. The reduction in maximal pupil diameter after administration of 0.0625% pilocarpine and the change in anisocoria from baseline to post-pilocarpine test in the 0.0625% group showed the best diagnostic performance for denervation supersensitivity in unilateral Adie's tonic pupil. The false positive rates of pupillary constriction after instillation of dilute pilocarpine were significantly higher in the 0.125% group than that in the 0.0625% group. The strength of our study is that pupillary examinations were objectively quantified with a dynamic digital pupillometer. We also provided a suitable cutoff value to be used in clinical practice.

In this study, false positive rates of the dilute pilocarpine test in the unaffected fellow eye were significantly higher in the 0.125% group than in the 0.0625% group. Normal pupil constriction could be detected after 0.125% pilocarpine instillation but was insignificant with a lower concentration of 0.0625% pilocarpine. Leavitt et al.^10^ reported the pupillary responses to four different concentrations of pilocarpine in normal subjects. They concluded that normal pupils constrict in the presence of 0.125% pilocarpine, and therefore suggested the use of 0.0625% pilocarpine to distinguish Adie’s tonic pupil from normal pupils^10^. Cohen et al.^8^ revealed that 0.0625% pilocarpine caused consistent miosis in a tonic pupil while only 0.5 mm or less constriction was found in the unaffected fellow eye, indicating the high specificity of the 0.0625% pilocarpine test. This is in line with our results in which only one subject experienced pupillary constriction of more than 0.5 mm in the unaffected fellow eye of the 0.0625% group.

Although the 0.125% dilute pilocarpine test is generally performed to confirm the clinical suspicion of a postganglionic parasympathetic lesion, the test's reliability to accurately distinguish Adie's tonic pupil from a preganglionic oculomotor nerve lesion has been questioned^10,17^. False positive pupillary constriction after 0.1% pilocarpine administration in dilated pupils has been reported in 15% of normal subjects and also in some preganglionic oculomotor nerve disorders^9,11,18^. While 0.125% dilute pilocarpine induces variable miosis both in the dilated pupil and the normal fellow eye, the amount of pupillary constriction is known to be more pronounced in the larger pupil^18^. Since the amount of pupillary constriction caused by 0.125% pilocarpine is variable depending on the baseline pupil size, this makes the interpretation of PLR results more difficult. This is consistent with our results showing a wide range of variability in the change of anisocoria after 0.125% pilocarpine instillation in the control group. Conversely, there is little variance after 0.0625% dilute pilocarpine instillation in the control group including preganglionic oculomotor nerve lesions. This explains the superior diagnostic value of 0.0625% pilocarpine for detecting cholinergic supersensitivity as compared to 0.125% pilocarpine.

In view of these findings, the use of 0.0625% dilute pilocarpine, instead of 0.125%, is strongly recommended for detecting denervation supersensitivity as a standard diagnostic test for Adie's tonic pupil.

Our study supports the validity of using dilute pilocarpine testing and digital pupillometry for diagnosing unilateral Adie's tonic pupil. Using digital pupillometry, we could provide objective criteria for detecting unilateral Adie's tonic pupil. The diagnostic accuracy was largest for maximal pupil size reduction and the change in anisocoria after administration of 0.0625% pilocarpine. The sensitivity was highest with a cutoff value of 0.5 mm or more reduction in the maximal pupil size and with a change in anisocoria ≧ 0.5 mm. Most of the previous studies concerning the efficacy of dilute pilocarpine test measured the pupil size on photographs or biomicroscopy^8,11,17^. However, the interpretation of pupil size and amount of anisocoria are influenced by ambient light conditions and are subject to inter-examiner variability.

In this study, we not only measured the effect of pilocarpine in each eye, but also compared the difference between both eyes. When assessing cholinergic receptor supersensitivity, it is of greatest importance to measure the change in anisocoria at darkness from baseline to post-pilocarpine. In the condition of darkness, the sphincter is inhibited, and the only effect that is measured from baseline to post-pilocarpine is the cholinergic effect. The light reflex complicates the assessment, because the effect of pilocarpine and that of light on the remaining innervated sphincter segments are measured together. After dilute pilocarpine instillation, the supersensitive eye will contract more than the unaffected fellow eye and it is this difference, the change in anisocoria, that is critical.

Pupillary constriction in response to dilute pilocarpine observed in the tonic pupil is explained by cholinergic denervation supersensitivity^19^. Unfortunately, the clinical utility of cholinergic supersensitivity for diagnosing tonic pupil is limited because it is absent in about 20%^20^. Therefore, additional findings of segmental palsy of the iris sphincter and accommodative paresis are important diagnostic clues of a postganglionic lesion of the parasympathetic nerve^1^. However, segmental palsy is not found in about 10% of Adie’s tonic pupil, and accommodative paresis may resolve over several months^1^. Consequently, objective interpretation of denervation supersensitivity is still a useful clinical tool for the diagnosis of Adie’s tonic pupil and differentiation from other causes of dilated pupils.

Our study has several limitations. First, normative data were not available for the PLR parameters obtained from digital pupillometry; however, we overcame this problem by comparing the data with the unaffected fellow eye. Second, the absence of supersensitivity does not necessarily rule out the diagnosis of Adie's tonic pupil^1^. In this study, we included patients with more than six weeks of symptom duration. As reinnervation by accommodative fibers becomes denser, receptors are downregulated to lose cholinergic supersensitivity^21^. It is usually not possible to know the exact time of onset, thus some of the patients may have already lost much of their previous supersensitivity and the degree of tonic pupil may have been variable^21^. Therefore, we confirmed that the degree of tonic pupil was not significantly different between the two dosage groups by comparing both the baseline anisocoria at darkness and the change in anisocoria from maximal pupil diameter at darkness to the peak contraction from light stimulus. Third, analyzing the dynamic changes of PLR as a function of time can provide more clear-cut evidence whether there is a real difference between each dosage groups. However, the portable digital pupillometry we used for the analysis provided only simplified graphs of the pupillary light reflex, so time-based analysis could lead to errors. Moreover, the main purpose of this study was to find a simple diagnostic tool that can be applied immediately in the clinical field. Therefore, despite its limitations, we picked the average pupil diameter among all PLR parameters. Finally, the control group was selected among patients with heterogeneous conditions, such as sphincter injury, oculomotor nerve palsy, and physiologic anisocoria. To confirm the efficacy of each concentration of dilute pilocarpine test for the diagnosis of unilateral Adie's tonic pupil, it was essential to compare the PLR parameters of patients with unilateral dilated pupils, and not that of normal subjects, in order to derive the ROC curve of the diagnostic ability of dilute pilocarpine tests.

In conclusion, we evaluated the diagnostic efficacy of dilute pilocarpine for detecting denervation supersensitivity in unilateral Adie’s tonic pupil. Pilocarpine at a 0.0625% dilution causes significant constriction of the affected pupil with minimal effect on the unaffected fellow eye and other causes of dilated pupils. Thereby, the 0.0625% diluted pilocarpine test is a reliable and objective method for the diagnosis of denervation supersensitivity in unilateral Adie's tonic pupil.

## Materials and methods

### Study subjects

We retrospectively reviewed patients who were diagnosed with unilateral Adie’s tonic pupil and underwent the dilute pilocarpine test between January 2011 and December 2019 in the Neuro-ophthalmology clinic of Seoul National University Bundang Hospital. Adie’s tonic pupil was diagnosed according to the following criteria: presence of anisocoria with unilateral absent or slow pupillary light response, normal ocular movement with segmental sphincter paralysis^22^. Two expert neuro-ophthalmologists (J-MH and HKY) confirmed the diagnosis of unilateral Adie’s tonic pupil.

Demographics and clinical characteristics were noted including age, sex, spherical equivalent refractive errors, best corrected visual acuity, duration of symptom, prior diagnosis from a primary clinic, age at diagnosis, presence of diabetic mellitus and hypertension, and any history of topical drug usage. Patients were excluded from the study if they had other neurologic abnormalities, congenital ocular anomaly, previous history of vitreoretinal surgery or ocular trauma, uveitis, Horner's syndrome, syphilis, or Guilin-Barre syndrome^4,19,23^. Patients were also excluded if they had any medical history of use of parasympatholytic and sympathomimetic drugs. To analyze inter-eye differences, subjects diagnosed with bilateral tonic pupils were also excluded from the study.

Patients who presented with anisocoria and unilateral dilated pupils that resulted from other causes were selected as controls. Some of these causes included physiologic anisocoria, oculomotor nerve palsy, sphincter injury, angle closure glaucoma, benign episodic pupillary mydriasis, and migraine with episodic unilateral mydriasis. Only subjects with a symptom duration of more than six weeks and less than three months were included. Both patients and control subjects underwent the dilute pilocarpine test with one of the two concentrations, 0.125% or 0.0625% pilocarpine.

### Pupillary light reflex measurements by digital infrared pupillometry

We used an automated monocular infrared pupillometer (PLR-200 pupillometry; NeurOptics Inc., Irvine, USA) to record the PLR^14,15^. Each subject was dark-adapted for three minutes before measurement. PLR was consistently measured from the right to the left eye. Patients were instructed to focus on a small target at least 1 meter away with the untested eye. The light stimulus was a white light with an illumination wavelength of 949 nm, intensity of 180 microwatts/cm^2^, and duration of 185 ms. The pupil size recording frequency was 32 frames per second, and the recording lasted up to 5 seconds. A trial rejection approach was used to minimize the risk of defining artifacts as actual pupillometry data^24^. If the pupillometer failed to collect a certain part of the pupil diameter change over time because of eye blinks, the measurement was discarded and repeated.

### Dilute topical pilocarpine test

Quantitative analysis of the PLR parameters and pupil photographs taken at baseline and after the pharmacological test is the standard examination protocol for patients with anisocoria in the Neuro-ophthalmology clinic of our institution. During measurements, the subject’s head was stabilized, and the subject looked at a red-light target at 1 m with the untested eye. The dilute topical pilocarpine test was performed with either a 0.125% (0.125% group) or 0.0625% (0.0625% group) concentration as described by Leavitt et al.^10^ Briefly, a sterile dropper containing 0.125% or 0.0625% dilute pilocarpine was prepared. Thirty-four of the 56 patients (60.7%) were administered with 0.125% pilocarpine and 22 (39.3%) with 0.0625% pilocarpine. Sixty-one control subjects were included for comparison, 28 subjects were administered with 0.125% pilocarpine and 33 patients with 0.0625% pilocarpine. The baseline PLR of each eye was measured twice in the dark using digital pupillometry. One drop of dilute pilocarpine was placed in each eye and PLR measurements were repeated after approximately 30–40 minutes using the same method as at baseline^10^. Topical anesthesia was not used during the examination.

### Statistical analysis

Statistical analysis was performed with R free statistical software (http://www.R-project.org, ver. 4.0.2, R Foundation for Statistical Computing, Vienna, Austria)^25^ and related packages, including ggplot2^26^, plyr^27^, and pROC^28^; and MedCalc Statistical Software version 19.0 (MedCalc Software, Ostend, Belgium). The comparison of PLR parameters and ocular characteristics between patients with unilateral Adie’s tonic pupil and controls was performed using the unpaired t-test and ANOVA analysis with post hoc test. Anisocoria was defined, for both groups, as the difference in pupil diameters between the affected and the unaffected fellow eye. The change in anisocoria at darkness from baseline to post-pilocarpine test was defined as the baseline anisocoria minus post-pilocarpine test anisocoria. For each PLR parameter, the value of the inter-eye difference was compared with the measurements of the control group. The diagnostic usefulness of changes in anisocoria and PLR parameters between baseline and post-pilocarpine test was assessed using the AUC. We included Bayesian analyses as a complement of the conventional null hypothesis statistical testing. The model with the highest Bayes factor is the most suitable model. In order to be considered meaningful evidence for the null or alternative hypothesis, the bayes factor should be greater than 3 or less than 0.33^29^ We used the BayesFactor package for R, which implements the methodology outlined in Rouder et al.^30^ Data are presented as means ± standard deviation.

### Ethical approval

This study adhered to the Declaration of Helsinki and the protocol was approved by the Institutional Review Board of Seoul National University Bundang Hospital (IRB No.: SNUBH B-1904/537-106). All clinical investigation was conducted according to the principles expressed in the Declaration of Helsinki. Patient records and information were fully anonymized and de-identified prior to access by any of the authors. The ethics committee waived the requirement for informed consent. Two patients provided informed consent to publish the images in an online open access publication.
